# ‘They Forget I’m Deaf’: Exploring the Experience and Perception of Deaf Pregnant Women Attending Antenatal Clinics/Care

**DOI:** 10.5334/aogh.2942

**Published:** 2020-08-07

**Authors:** Olufemi T. Adigun, Thanduxolo P. Mngomezulu

**Affiliations:** 1 University of Zululand, ZA

## Abstract

**Background:**

Antenatal care (ANC) services provide access to integrated health management for several pregnancy related conditions. Unfortunately, deaf pregnant women remain vulnerable during pregnancy due to lack of access as well as communication barriers at antenatal clinics in Nigeria.

**Objective:**

The primary aim of this study was to explore the experiences and satisfaction of pregnant deaf women with antenatal care in Nigeria.

**Methods:**

This was a qualitative study, conducted among nine deaf pregnant women from two local government areas, attending both private and public health facilities for antenatal care in Ibadan, Oyo State, Nigeria. Data were collected using semi-structured, video recorded one-on-one interviews, with sign language as the medium of communication. The interviews were conducted until saturation of the themes was reached. The recorded interviews were precisely transcribed and thematic analyses were conducted on the data obtained.

**Findings:**

The mean age of the participants was 29.5 years. Participants indicated that they had registered/booked for antenatal care in their second trimester. Registration at this stage was regarded as late registration of the pregnancies. Communication difficulties during their ANC (antenatal care) visits, distance and location of the clinics, knowledge and perception of what ANC entailed, finance/cost, and health care professionals’ attitudes towards the participants were the major themes identified for late ANC bookings. Participants who attended privately owned health care facilities for ANC had more satisfaction with ANC care than those attending publicly owned health facilities.

**Conclusions:**

Deaf pregnant women were knowledgeable about ANC but registered late for the service, largely due to communication difficulties, distance to the clinic, cost, and the perceived attitudes of the health care workers. There existed a variance in the level of satisfaction of deaf pregnant women who attended private or public health facilities.

## Introduction

Among African nations, the rate of maternal mortality in Nigeria remains worrisome. Besides India, Nigeria records the second highest number of annual maternal mortality [1,2]. However, statistics concerning maternal mortality among deaf women in Nigeria are unavailable. Deaf pregnant women, by the nature of their hearing loss and inability to actively respond to auditory stimuli, may be highly susceptible to greater risks associated with maternal and/or child mortality. Although evidence-based research on pregnancy and maternity experiences of deaf women is scarce, especially in Nigeria, there exists a plethora of research devoted to non-deaf pregnant women, with no attention paid to the ‘silent’ communities of deaf women [16,19]. Hence, this study aimed at exploring and elucidating the experiences and perceptions of deaf women attending antenatal care (ANC) in Ibadan, Nigeria.

Antenatal care is a programme designed to inform and educate expectant mothers about the status of their health and that of their unborn babies; to identify at-risk pregnant women through adequate and regular observation throughout the gestational period. According to the World Health Organization [WHO], the objectives of ANC are to offer consistent check-ups, examinations and screenings by doctors and midwives to facilitate prevention, detection, and treatment of potential health and pregnancy complications such as pregnancy induced hypertension, anaemia and malaria [3]. During the gestational period, the foeti and mothers are expected to be clinically assessed. Mothers should receive useful information about health services, exercises, and nutritional maintenance in order to prevent the development of pregnancy-related complications and to promote easy and safe delivery at birth. Hence, antenatal care in Nigeria is a platform established in accordance with the World Health Organization’s systemic review to educate and provide information to expectant mothers about the need for regular check-ups [4]. These check-ups by midwives and doctors will prevent, detect, and attend to potential pregnancy-related complications, irrespective of the mother’s disabilities. In order to achieve positive motherhood experiences, booking for antenatal care is recommended in the first trimester of the gestational period [5].

Existing studies have shown variations in the initiation of ANC among expectant mothers in Africa [6,7]. A few of the expectant mothers in developing countries sought early initiation of ANC, while a larger percentage of the mothers presented themselves late for ANC [7,8,9]. Studies have associated this late initiation with long distances or travel times to health centres, lack of health insurance, poor socio-economic status or financial constraints, low levels of education, parity, inadequate knowledge of the importance of antenatal care, unwanted pregnancy, previous use of contraceptives, employment status, cultural norms and ethnicity [6,7,10,11,12,13]. Unfortunately, a close examination of the findings from previous studies revealed that none adequately examined the role of a disabling condition on ANC initiation times. In terms of the current study, the disabling condition among pregnant women is, specifically, deafness.

Irrespective of when the antenatal care was initiated, studies among pregnant women attending antenatal clinics – whether with disabilities or not – noted that: Satisfaction with ANC, discriminatory attitudes and inequalities, inadequate infrastructure, awkwardness and insecurity, violation of individual limitations, lack of adequate support systems, lack of confidence in the parenting ability of disabled women, and problems communicating with health professionals were some of the contentious issues related to women receiving ANC [14,15]. Additionally, recent studies noted a significant disparity and inequality in access to antenatal care services among women from developing nations [16,17,18]. A closer look at the inequalities in access to antenatal care service suggested that this gap was wider for women with disabilities, particularly deaf pregnant women. O’Hearn, in a comparative study on the experiences of deaf and hearing pregnant women attending antenatal care, reported lower overall quality and satisfaction with antennal care, poor communication with midwives and physicians, and less antenatal clinic attendance by deaf pregnant women due to the absence of sign language interpreters, in comparison to the pregnant hearing women within the same health care facility [19]. While sign language interpretation service remains an important element in effective two-way communication between the deaf and hearing, available evidence suggests that lower satisfaction with services and lower utilisation of reproductive health information exists among deaf individuals [20,21]. While much of the information given during antenatal visits is largely presented verbally, deaf women whose sense of hearing not functional enough to stimulate sound signals are deprived of essential antenatal information. Therefore, it is assumed that deaf women whose disability can be considered ‘silent’ may be highly marginalised and excluded from accessing adequate reproductive health information from midwives and other health practitioners, since communication modes are not in sign language. There is a disconnection in communication between ‘patients’ and ‘health practitioners,’ which may alter the perception of deaf pregnant women about the disposition of health practitioners towards them. Ultimately, the perception and experience of pregnant deaf women during antenatal care may be hampered by the lack of adequate information received, and by their dissatisfaction compared to non-deaf pregnant antenatal attendees. Regrettably, despite the large body of research that has investigated and explored the perceptions and experiences of non-deaf pregnant women, studies on deaf pregnant women seeking antenatal care and services, especially in developing countries such as Nigeria, are scarce [23].

The motivation-facilitation theory of prenatal care access stresses the dynamic interplay of a woman (deaf pregnant woman) and the clinic (privately or publicly owned) and encourages health care workers to facilitate access and effective utilisation of ANC by actively improving the clinical environment [24]. Using this theory, the current study explored the experiences and satisfaction of pregnant deaf women who received antenatal care in Ibadan, Oyo State, Nigeria in an attempt to provide answers to the following critical questions:

- At what stage of pregnancy development did the participants register for antenatal care?
- What were the factors responsible for the timing of the participants’ registration for antenatal care?
- Were deaf women satisfied with antenatal care and the services received in the study’s location?

## Method and materials

### Study design and setting

The interpretivist paradigm and a qualitative descriptive research design were adopted for this study in order to explore the views of deaf women about their experiences during antenatal care in Nigeria. The qualitative research design was considered suitable for this study because it aimed at exploring the subjective experiences of deaf women who had registered for antenatal care in Ibadan, Oyo State, Nigeria. Ibadan is the capital of Oyo State and is located in the South West region of Nigeria. Ibadan is predominantly a Yoruba speaking city but has some other tribes, such as the Igbos and Hausas, who migrated to the city from other parts of Nigeria. The use of a qualitative research design was considered appropriate for this study because it was the research approach that allowed for the phenomenon to be studied in its natural environment based on beliefs, aspirations, values, motives and attitudes of the participants in relation to space and their social interactions [25].

### Procedure

Ibadan, Oyo State, Nigeria was purposively selected as the study area because of its longstanding history of a deaf cluster, and because of the authors longstanding relationship through research studies and social interaction with the deaf communities in Ibadan [26,27,28]. The Ibadan metropolis consists of five local government areas, namely Ibadan North, Ibadan South West, Ibadan North West, Ibadan North East and Ibadan South East. Two local government areas (Ibadan North and Ibadan North East) from the Ibadan metropolis were then randomly selected through a simple random sampling technique using a balloting system. The authors then contacted the chairpersons of the local deaf associations, with the assistance of the State Chairperson of the Deaf Women’s Association of Nigeria (DWAN). These local chairpersons further introduced the researcher and his research assistants to deaf women who had previously registered for and attended antenatal clinics.

A purposive sampling procedure was also employed to select nine pregnant deaf women, comprising eight Yoruba women and one Igbo woman, who primarily used sign language as a means of communication (Yoruba and Igbo are two of the three dominant ethnic groups in Nigeria). Recruitment of the participants was conducted using the WhatsApp short messaging service and face-to-face invitations. Nine deaf women met the inclusion criteria of this study which were: (i) Participants had to communicate primarily through sign language; (ii) they had to be of reproductive age (18–45 years); (iii) they had to have registered for and attended antenatal clinics in the six months prior to the study period; and (iv) they had to be willing to voluntarily participate in the study.

### Data collection

A semi-structured, video-recorded, one-on-one sign language interview was conducted by the researcher and the research assistants with each of the nine participants in their homes between December 2 and December 28, 2019. The focused and discursive interview allowed the participants and the researcher or research assistants to explore issues of ANC for deaf pregnant women. Due to the nature of the participants, the researcher was flexible and did not control the responses, actions, pantomimes or gestures of the participants interviewed. The researcher prepared an interview guide to determine the experiences and challenges faced by the deaf women during their antenatal clinic visits in Ibadan. The advice of Cohen, Manion and Morrison on interview procedures was adopted [29]. The interview guide obtained some demographic information such as the respondents’ age, marital status, the number of full-term pregnancies, and the hearing status of their spouses. The interview guide also contained specific questions about their experiences, such as: At what stage of pregnancy did the respondents register for antenatal care? What were the respondents’ greatest fears and/or challenges while receiving antenatal care? How would the respondents describe the attitudes of the midwives and the other health professionals towards them during their antenatal care visits? The interview sessions lasted for about 35 minutes each.

### Ethical consideration

Participants were duly informed about the aim of the study by a trained research assistant who was proficient in sign language. In line with the ethics of research, the research assistant presented each participant with a printed consent form written in the English language. The content of the consent form was explained to each participant by the research assistant, using sign language. Once adequate understanding was ensured, each participant completed and appended their signature to the consent form. Participants were assured of the confidentiality of their profiles and responses. Permission to conduct this study was sought and received from the executives of the Deaf Women Association of Nigeria (DWAN), Oyo State branch. The study thus adhered to ethical considerations in research.

### Data analysis

Recorded video interviews were transcribed, coded, and anonymised. The research assistant who transcribed the video content agreed to the ethics of research *vis-à-vis* the confidentiality of the interviewees. In order to ensure confidentiality and anonymity, a unique identifier was given to each study participant, to be used instead of their names. Participants in the study were thus represented as DW1, DW2, DW3, DW4, DW5, DW6, DW7, DW8 and DW9. The authors cross-validated the transcribed documents with the video interviews before analysing the data. The authors, with the assistance of the research assistants, modified and reconstructed the English of the transcriptions of the recorded interviews given by the interviewees, to better reflect structured statements in terms of grammar, suitable for further analysis. For instance, DW3, using sign language, said: “Me go clinic antenatal 6 month pregnancy.” This was corrected and coded as: “I visited the clinic for antenatal at my sixth month of pregnancy.” A thematic content analysis was performed on the transcribed interviews. The goal of using the thematic analysis was to identify recurring themes in the interviews, in order to use the themes to describe and address the research questions. The interviews were coded and organised using the repetitive themes from the transcribed documents. The iterative process of the analysis, comparison, and summarisation of the data collected ensured that the research questions could be answered and that a conclusion could be drawn.

## Results

The youngest participant in this study was 22 years old, while the oldest was 37, and the mean age of the participants was 29.5 years. Five of the participants were married, of them four had deaf husbands while one was married to a hearing husband. Only three of the participants had not been using contraceptives prior to becoming pregnant, while the other six indicated that they had been using contraceptives prior to their current pregnancies. Two of the participants of this study had five prior full-term pregnancies; four of them had between three and four pregnancies, and only two participants were on their first or second pregnancies. Two of the study participants had a tertiary education while three had a basic education, three had a secondary education and one had no formal education at all. Two of the nine participants reported a net monthly income of greater than 50,000 naira (about $200), five had a monthly net income ranging between 10,000 to 50,000 naira (less than $200) and two had a net monthly income of less than 10,000 naira (less than $100). Furthermore, two of the nine participants had registered for ANC at a privately-owned hospital, while the others had registered for care at public health facilities.

## Qualitative data analysis

This study analysed the recorded video interviews of nine deaf pregnant women seeking antenatal care and services in Ibadan. The nine participants were at different gestational stages of their pregnancies. The researcher prompted the participants to respond to **research question one** with the use of sign language, to elicit information on when they had registered for antenatal services. Based on their responses, the common theme was that the majority of the participants had started visiting antenatal clinics during the second trimester of their pregnancy, while only one participant had begun seeking antenatal care during her first trimester. One participant had this to say:

*For all my two children and this current pregnancy, I usually register for antenatal when the pregnancy is advanced. Hmmm, (Shaking her head to get a precise date) around 6 to 7 months of pregnancy* (DW3, 34 years of age).

Another participant agreed that she had started her antenatal care at a later stage, for reasons known only to her:

*I prefer to go late to the hospital for antenatal care, at about 5 months of pregnancy. I registered for antenatal care at the health centre about 2 months ago* (DW7, 28-year-old with second baby due about two months after the interview).

Contrastingly, one participant declared that her antenatal clinic visits had started during the first trimester of her pregnancy, at about eight weeks after her last menstrual period (LMP):

*I went to the clinic to register for the antenatal last month (referring to November 2019)* (DW8, 22-year-old recently married to a deaf man).

**Research question two** sought to identify the factor(s) that determined why they had registered their pregnancies and sought antenatal care at the particular stages indicated in response to research question one. The key themes were categorised based on the participants’ views, perceptions, experiences, and concerns raised about the antenatal booking process and their appointments. Their explanations are discussed under two themes, namely (a) personal factors, and (b) health system related factors.

### (a) Personal factors

The personal factors associated with the time of their antenatal registrations have been conceptualised in this study as variables individual to the interviewees before and after their pregnancies. Hence, based on the reoccurring themes which stemmed from the interview, personal factors associated with the timeframe of the antennal registration have been further subdivided into three themes; namely (i) distance and location, (ii) knowledge and perception, (iii) finance/cost.

#### (i) Distance and location

Seven participants reported that the distance from their homes to the hospital was a major factor that prevented them from going to register for antenatal care earlier than they had. Two of the interviewed deaf pregnant women lived in remote areas in the Ibadan North East local government area.

One of the participants had this to say:

*The distance from my house to the main road where I will board a taxi to the hospital is far. Taxis cannot come into my area; it is only ‘okada’ (motorcycles) that comes into my area to take people to the main road. Many times, due to the bad road, people used to fall from the Okada, especially when it rains, and the road is slippery. I can’t afford to climb on the motorcycle when I am pregnant. So, I waited until I was about 6 months pregnant before I went to register for the antenatal* (DW1, 29-year-old).

Another participant corroborated the claim of DW1:

*The state hospital where I registered for antenatal care during my first pregnancy was close to our former residence. I am registered at the same place now, but it is far from where we live presently. I will have to take three different taxis before I get to the hospital. I get more frustrated when there is traffic congestion…and you know I will be quite tired when I do such. So, that was why I thought that going early to register for the antenatal care will add to the stress of early stage of pregnancy so I had to delay till I was strong enough at 5 months* (DW7, 28-year-old).

#### (ii) Knowledge and perception

All the participants unanimously attested to the fact that they were fully aware of the need for antenatal care for pregnant women. Also, they understood the importance of attending antenatal clinics but their perception of the activities or type of care during the antenatal visits differed. For instance, two of these participants stated that:

*I have always known that a pregnant woman usually goes to the hospital for check-ups in order to ascertain the health status of the baby* (DW4, 27 years old).

*I know that going for antenatal visits is good. The doctors will check and confirm how the baby is growing or developing and they will give pregnant women some important information about their health and that of the baby. The nurses will sometimes advise those who attend antenatal care about the type of food to eat* (DW6, 36 years old).

Additionally, the researcher inquired about their knowledge of the availability of antenatal care, and what their reasons were for the late registration for antenatal services in their second trimester.

Participant DW2, a 37-year-old deaf woman who was carrying her fifth child, stated that:

I didn’t know I was pregnant; I was using contraceptives and still having my menstrual flow. I knew about the pregnancy at about 4 months and some weeks. I was devastated because I don’t want any more children. I was not motivated to go out of my home to talk of registering for antenatal.

The researcher further probed about their perception of antenatal care for deaf pregnant women and identified that they were bothered about communication issues during their antenatal visits. All declared that they had always found it difficult to interact with other attendees or health professionals during their antenatal care visits. All the participants indicated that they had tried to lip read, had written on pieces of paper or had just followed what others were doing. Participant DW2, a 37-year-old mother of four children, stated that:

It is not easy for deaf pregnant women who attend antenatal care, especially at a hospital where there are many non-deaf pregnant women. This is because the deaf pregnant women will be frustrated because, without the help of a sign language interpreter, she will not benefit from all the lectures that the midwives normally give.

Another participant had this to say:

*I normally go to the health centre with my notepad and pen because I communicate with the nurses and doctors through writing. Sometimes, some of the nurses and doctors will try to describe something that I may not understand through gestures, pantomime or even dramatization* (DW5, 25-year-old who attended an antenatal clinic at a privately owned facility).

However, another participant indicated that her husband always accompanied her to make communication easier for her:

*During my first pregnancy, I usually go with my husband (hearing) who stands by me to interpret all needed information to me. In fact, he was there in the labour room with me. But now, since I understand the nature and structure of antenatal care and services, I usually lip read and ask either the midwives, doctors or other antenatal attendees to communicate with me through my pen and note pad* (DW9, 30-year-old civil servant).

All nine participants in this study unanimously stated that communication methods with deaf pregnant women in health facilities providing antenatal care in Ibadan were not appropriate. According to DW6 (36 years old) and DW2 (37 years old):

There is yet to be a health facility in Ibadan with a standby sign language interpreter for the sake of the deaf patients. Any deaf patient would either go with his/her own sign language interpreter or make use of paper and pencil.

#### (iii) Finance/cost

The majority of the deaf pregnant women who participated in this study identified the cost of attending an antenatal clinic as a critical barrier to their early registration and/or their utilisation of ANC services. Hence, the low socio-economic status of the participants was identified as a major theme around the finance/cost of the services rendered at antenatal clinics. This participant expressed that:

*Like I said earlier, the health centre is too far from my house, I will need about three taxis before I get to the hospital… and again, I and my husband don’t have good jobs and the financial strength to take care of our financial needs…we are just managing. That’s one of the major reasons why I delayed my registration for antenatal until when the pregnancy was 6 months.’* (DW7, 28 years old).

Similarly, another participant stated that:

*The cost of registration at a nearby privately-owned hospital is [too] much, so I have to travel a long distance to the state hospital because it is cheaper there, but I still have to take an interpreter with me to every antenatal visit. So, the additional cost of [a] sign language interpreter, transportation, ultrasound scans, and all other requirements for antenatal registration is too much for me, considering my monthly income of 20,000 naira (less than $100)* (DW4, 27 years of age).

### (b) Health system related factors

The role of the health care system is vital for the nurturing and care of pregnant women, in terms of achieving sustainable development goals. This study sought to determine why deaf pregnant women in Ibadan registered their pregnancies and only sought antennal care at a late stage in their pregnancies. The findings revealed that a non-inclusive health system for this group of people contributed to their late attendance at antenatal clinics. Regarding the health system related factors, the participants identified two major deterrents to accessing antenatal care. Communication issues with health care workers, and the attitudes of the health care professionals towards deaf women while providing antenatal care were the two major themes identified in this study.

#### (i) Communication issues during ANC visits

Communication and information dissemination by midwives, doctors, and other health care professionals while providing antenatal care was primarily done verbally. Unfortunately, verbal communication was a mode of information dissemination which did not favour these individuals who are deaf. Communication between the deaf pregnant women who were interviewed in this study and the other ANC attendees, as well as with the health care professionals in the clinics, was a serious challenge for them. Eight out of the nine deaf pregnant women interviewed stated that they had never been comfortable with the volume of information received during ANC visits. In other words, a communication and information gap existed between the deaf pregnant women and the health care professionals in Ibadan hospitals where ANC services were being rendered. One participant affirmed that:

*I don’t feel good when midwives or doctors disseminate information on antenatal days, and I can’t fully understand what I’m supposed to* (DW8, 22 years old).

Another participant added this:

*Most of the time, nurses at the state hospital where I registered for my ANC forget that I am deaf; most times, they shout at me when they pass instructions to all pregnant women and I am just there without following their instructions* (DW6, 36 years old).

#### (ii) Attitudes of the health care professionals

Seven out of the nine deaf pregnant women who participated in this study were not comfortable with the attitudes of the health professionals who attended to them during their ANC visits. These seven participants were not comfortable with the reactions of the nurses or midwives towards them at every ANC visit. One of the participants stated that:

*Truly, health facilities and health personnel are overburdened due to excess work and overcrowded antenatal waiting rooms, but they discriminate against the deaf* (DW2, 37 years old).

Another participant reinforced this theme:

*Many times, they are usually partial against me, simply because I’m deaf. They will tell me to wait until after they have attended to others who either came with their husbands or who are rich* (DW4, 27 years).

The concerns of these participants revolved around the attitudes of the nurses: Stereotyping and labelling. For instance, one participant, while dramatizing the behaviour of the nurses at a public hospital, lamented:

*… The nurse will call you ‘aditi’ (‘Aditi’ in the Yoruba language means a deaf person). My sign language interpreter told her several time to call me by my name and not ‘aditi’. The nurses sometimes scold and ridicule you in front of others and make you look stupid* (DW3, 34 years old).

However, another participant applauded the nurses at her privately-owned clinic where she had booked for her antenatal care:

*… I had the mind-set that nurses are rude and wicked, so I was nervous to come to the clinic. Fortunately, the nurses attending to me have been very nice and patient with me. They try their best to listen and to understand my expressions when I don’t write* (DW5, 25 years old).

In response to **research question three** which assessed how satisfied these deaf pregnant women were with services rendered at the antenatal clinics in Ibadan, the participants provided the following feedback: Two deaf pregnant women who had registered for their ANC at a privately-owned hospital expressed a high level of satisfaction with the conduct and services of the health professionals in those hospitals.

*I am very happy with where I registered for my ANC; the nurses there are very accommodating and always caring. They attend to me very well any time I go there. I am very satisfied with their services* (DW8, 22-year-old).

However, the other seven participants in this study who accessed ANC at public health facilities expressed dissatisfaction with the services rendered by the health professionals from the various health facilities:

*Whenever I go for my antenatal care, especially if my sign language interpreter is not with me, I feel very unhappy with things done at the clinic. I get disturbed with the action of the nurses/midwives. They will be shouting at me and making jest of me. I often see them laugh with reckless abandon at me. Seriously, if I have money, I would have changed to a privately-owned clinic for the ANC* (DW3, 34 years of age).

*They* (referring to nurses and midwives) *don’t attend to the deaf like they do to those who are not deaf. They discriminate against the deaf. I am not satisfied with their attitude* (DW6, 36-year-old).

## Discussion

Issues with disabilities as well as pregnancy remain one of the public health concerns for every government and developmental agency across the globe. Concerns about facilitating psychosocial well-being, integration, and social inclusion of persons with disabilities is yielding the desired results; deaf women have continued to enjoy a good family life, with many currently accessing antenatal care and services across the globe. However, many of those interviewed in this study revealed that they had only registered for antenatal care in their second trimester. Amongst the nine participants, seven stated that they had registered late for their ANC. Registering a pregnancy and booking for antenatal care in the second trimester of pregnancy has been deemed an unsafe practice or attitude by pregnant women; unfortunately, the findings in this study showed that deaf pregnant women often registered late for ANC, in line with the findings of other studies [4,5,30,31,32]. This study showed that several factors were responsible for the late antenatal registration by deaf pregnant women. Two major themes were identified as the determinant factors for the late initiation of care, personal factors and health system related factors.

The personal factors influencing late antenatal care initiation among deaf pregnant women were observed to include: The distance from and location of the clinics, knowledge and perception of the care offered, as well as the financial costs incurred when travelling to the clinics. The participants in this study were concerned about the distance covered to receive their antenatal services. In other words, deaf pregnant women usually travelled long distances to access ANC, thus the distance travelled remained one of the major factors for late initiation of ANC. This study appears to be the only one to have identified the role of distance to be travelled as a factor responsible for deaf pregnant women delaying seeking antenatal care. Studies among non-deaf pregnant women have, however, linked late registration of pregnancy and initiation of ANC to distance to health facilities [5,32,33,34].

Unlike the studies which reported that lack of knowledge contributed to late ANC initiation, this study confirmed that deaf pregnant women were knowledgeable about the importance of seeking care and the services offered at antenatal clinics [7,32]. In fact, one of the participants affirmed this with the following statement: “I know that going for antenatal visits is good. The doctors will check and confirm how the baby is growing or developing and they tell pregnant women some important information about their health and that of the baby.”

The findings from this current study supported Finlayson and Downe, who maintained that pregnancy awareness and disclosure could contribute to the timeframe for ANC initiation [35]. Furthermore, this study revealed that the lower socio-economic status of deaf women contributed to their late registration at antenatal clinics. Due to their meagre monthly income and their financial responsibilities, the participants in this study stated that they had registered their pregnancies and sought ANC later in their pregnancies in order to reduce costs and to adequately attend to other pressing financial responsibilities. Earlier studies have reported similar findings among non-deaf pregnant women in Ibadan, Nigeria [7,12,36].

Participants reported that communication difficulties during their antenatal visits and the perceived negative attitudes of the health care professionals towards them were factors that influenced their decisions to seek care later in their pregnancies. This implied that ineffective two-way communication and the interaction between the deaf patients and the midwives and/or their physicians had negatively impacted their experiences at the clinics. This finding contradicted those of previous studies where discrimination, inequalities, awkwardness, insecurity, a lack of confidence in the parenting abilities of disabled women by health care workers, and inadequate support systems were the major challenges faced by women with disabilities when accessing qualitative health care services, including antenatal care [14,15,16,17]. Deaf women who participated in this study stated that they relied either on writing or on hiring sign language interpreters for communication when they visited antenatal clinics. Unfortunately, Gichane et al. noted that it was unethical for deaf patients to make provision for interpreters as there was then no room for confidentiality of the information provided [16]. The current study’s finding was consistent with previous findings which noted that poor communication with health care professionals was a major difficulty experienced by the deaf population when using health care services [16,22,26,37].

Furthermore, this study revealed that the deaf women attending antenatal clinics, especially those located in public health facilities, expressed concerns about the negative attitudes of the health professionals. The participants were not comfortable with the attitudes of the nurses or midwives towards them during their ANC visits. Hence, the previous experiences of poor communication and quality of the interactions between the health care workers and these deaf pregnant women may have resulted in them initiating antenatal care late into their next pregnancies. In a similar study by Kaswa et al. among 20 IsiXhosa speaking pregnant women from the Eastern Cape, South Africa, it was reported that insensitivity, rudeness and poor attitudes of the health care workers often discouraged pregnant women from attending antenatal clinics [5]. The attitudes of health care workers that were insensitive to the nature and needs of patients significantly contributed to poor utilisation of antenatal services. This corroborated the study of Banda et al., where they submitted that weak, under-resourced, unsupportive health systems, as well as poor readiness of health care workers and facilities could negatively influence the perceptions of pregnant women about the quality of the antenatal care received in such facilities [38].

The attitudes of health care workers towards deaf pregnant women are a major factor that will shape their perceptions of and satisfaction with antenatal care. Although there are a dearth of studies that have assessed the satisfaction levels of deaf pregnant women attending ANC clinics, available evidence obtained from non-deaf patients at ANC clinics suggests that attitude, respect, sincerity, courtesy and healthy interpersonal relationships determine maternal satisfaction [39,40]. In line with the findings of earlier reported studies by Jallow et al. (2012) and Akodu et al. (2017), the participants in this study who accessed ANC care in public hospitals expressed a lower level of satisfaction with the antenatal services received. Jallow et al. rated the satisfaction level of pregnant women attending private antenatal clinics at 12% higher than those who sought care at public clinics [41]. The study among pregnant women from Lagos, Nigeria by Akodu et al. observed a 65.1% satisfaction level with ANC amongst the pregnant women who had registered for ANC at private clinics and only a 37.3% level of satisfaction for those who had attended public clinics for the purposes of ANC [41].

## Conclusion

This study has further advanced the knowledge about antenatal experiences and perceptions by bringing to the fore the experiences of deaf pregnant women in Ibadan, Nigeria. The findings from this study showed that deaf pregnant women only registered for antenatal care in their second trimester, predominantly as a result of communication difficulties and perceived poor attitudes of health care workers towards them. Deaf pregnant women attending privately-owned clinics for ANC were more satisfied with the antenatal services received than those who attended public health facilities. This variance in the level of satisfaction by deaf pregnant women who attended private or public health facilities for their ANC was also presumed to be influenced to a large extent by the socio-economic status of these deaf pregnant women.

### Recommendations

Based on the findings of this study, it is suggested that:

1. Deaf women should be encouraged to register for ANC in their first trimester of pregnancy through various orientation and advocacy programmes. Sign language should be used to communicate the importance of an early ANC booking to them.
2. Regular training/workshops on sexual and reproductive health for deaf adolescents and young adults should incorporate and emphasise the importance of early ANC initiation in their curricula. Such training should include audio-visual instructional materials that show the processes and activities expected of a pregnant woman who visits an antenatal clinic. Such gestures will prepare them ahead of antenatal visits by informing them of what is expected of them when seeking antenatal care and advising them of the role of health care workers at the antenatal clinics.
3. Each health care facility should make provision for sign language interpreters, especially those in Ibadan, Oyo State, where there is a cluster of deaf people. The facilities should employ at least two sign language interpreters in order to ease communication challenges between deaf patients and health care workers, and to promote the confidentiality of the information provided by the health care workers to deaf patients. The provision of sign language interpreters in each health care facility will not only promote the ethics of health care, but also reduce the financial burden on deaf pregnant women who have to pay for the services of sign language interpreters. Lastly, employing the services of sign language interpreters in public clinics will elevate the satisfaction levels and raise the confidence of deaf pregnant women who visit public clinics for ANC.

### Limitation of the study

This study was not exhaustive as it only engaged a qualitative research approach and only explored the experiences and perceptions of nine deaf pregnant women in Ibadan, Nigeria. This study did not investigate the construct using a longitudinal and quantitative study approach, nor did it access information from health care workers with respect to deaf pregnant women who visited their clinic for ANC. On this note, it would be expedient for future studies to consider further research which would incorporate the perceptions and experiences of health care workers with deaf pregnant women. Additionally, a longitudinal study using a quantitative approach to ensure a larger sample of participants is suggested.
